# Reduction of the Livestock Ammonia Emission under the Changing Temperature during the Initial Manure Nitrogen Biomineralization

**DOI:** 10.1155/2013/825437

**Published:** 2013-12-23

**Authors:** Rolandas Bleizgys, Indrė Bagdonienė, Ligita Baležentienė

**Affiliations:** ^1^Institute of Energy and Biotechnology Engineering, Aleksandras Stulginskis University, Kaunas distr. 53361 Akademija, Lithuania; ^2^Institute of Ecology and Environment, Aleksandras Stulginskis University, Kaunas distr. 53361 Akademija, Lithuania

## Abstract

Experimental data were applied for the modelling optimal cowshed temperature environment in laboratory test bench by a mass-flow method. The principal factor affecting exponent growth of ammonia emission was increasing air and manure surface temperature. With the manure temperature increasing from 4°C to 30°C, growth in the ammonia emission grew fourfold, that is, from 102 to 430 mg m^−2^h^−1^. Especial risk emerges when temperature exceeds 20°C: an increase in temperature of 1°C contributes to the intensity of ammonia emission by 17 mg m^−2^h^−1^. The temperatures of air and manure surface as well as those of its layers are important when analysing emission processes from manure. Indeed, it affects the processes occurring on the manure surface, namely, dehydration and crust formation. To reduce ammonia emission from cowshed, it is important to optimize the inner temperature control and to manage air circulation, especially at higher temperatures, preventing the warm ambient air from blowing direct to manure. Decrease in mean annual temperature of 1°C would reduce the annual ammonia emission by some 5.0%. The air temperature range varied between −15°C and 30°C in barns. The highest mean annual temperature (14.6°C) and ammonia emission (218 mg m^−2^h^−1^) were observed in the semideep cowshed.

## 1. Introduction

Ammonia (NH_3_) emissions compose significant environmental pollution, which is related to agriculture, particularly with husbandry [1]. The highest NH_3_ emissions of 75% derived from livestock production in dependence of livestock housing buildings, manure storage, and so forth [2].

Construction of the uninsulated (field climate) barns constitutes the principal livestock barn development trend in Lithuania. Furthermore, the conventional insulated barns are reconstructed to deep bedding/litter and box types, featuring uninsulated outer walls or only roof thermally insulated leading to the more natural cow housing conditions. Increasing productivity per cow and also altered perception of their housing conditions had a significant impact on such cowshed development trends. The recommended temperature has generally decreased. Epinatjeff [3] found out that the preferred temperature should range in between −10°C and +5°C for the cow which produces 20 and more kg of milk per day. Nonetheless, in order to maintain continuous manure removal, the barn temperature should not fall below −18°C [4]. Uninsulated barn type is well acknowledged across many countries. Thus, the intensive construction of suchlike barns was initiated in 1980s Western Europe, in 1990s in Scandinavian countries, Estonia and Lithuania [5, 6].

The modernization of the livestock buildings in Lithuania often involves some issues related to ambient air pollution: application of littered or unlittered livestock keeping technology; barn ventilation intensity; temperature maintained in the barn. The lack of reliable data on ambient air ammonia pollution from newtype cowsheds complicates the evaluation of these new technologies in terms of environmental protection. Thus, livestock is an important source of air pollution, which contributes to some 90% of the global ammonia environmental emission [7]. In agriculture sector, the most significant share of ammonia (50%) is emitted from cattle, 20–22% from pig, 7–21% from poultry, 9–18% from mineral fertilizing, and only 3–9% from horse, sheep, and other animals. However ammonia emission from barns composed 37–50% of total livestock emission [8, 9]. In order to reduce these emissions, it is necessary to apply sustainable technologies there. Though considerable investigations were carried out, the determination of the emissions from the open cattle barns still remained a problematic issue[10, 11]. Numerous researchers identified many methodological problems related to analysis of the gas emissions in open cattle barns. In addition, it is difficult to determine the accurate intensity of the ventilation in open barns. Therefore many studies attempted to improve research methods continuous measurements [12, 13]. Thereafter, intermittent measurement method was proposed to shorten the investigation period. Primarily, it is necessary to determine the annually changing microclimatic factors of a barn, for example, outdoor and barn temperature, ventilation intensity which varies throughout the year and affects the emission intensity, and select periods of measurements. Having measured the emission intensity, the significant annual emission from a barn can be forecasted [14, 15]. Fast and accurate determination of the gas concentration is also aggravated by the high ammonia concentration gradients within 0.1 m height above the manure layer [16]. Therefore, in order to obtain consistent data in emissions from manure, application of precise description of processes affecting the NH_3_ emission intensity is essential, notwithstanding permanent change of multiple factors (the changing air velocity, turbulence, and temperature) causes the ammonia emission to vary significantly affecting emission [2, 17]. Ammonia emission changes also significantly depended on the crust formation on manure surface. Crust formation is substantially influenced by the straw and dry matter content in the manure as well as environmental climatic conditions [18]. Besides, ammonia emission from cows is influenced by feeding during lactation [19]. When analysing the emission processes, the investigations are often limited to one of the most important and influencing factors affecting the emission intensity. The temperature can be treated as a key factor due to its higher positive impact on ammonia emission if compared to that frequency of manure removal, floor condition and cleaning, feeding cow activity. Thereafter, daily and annual ammonia emissions from manure vary unevenly. When temperature rises from 2°C to 20°C in the barn, the emission of ammonia pollution increases from 10 to 30 g per cow place (cow producing 1000 W total heat) per day [20]. Consequently, temperature decrease presents a good and effective way to reduce NH_3_ emissions. Moreover, the application of chemical agents is also proposed as a mean to reduce emissions from manure at high temperatures [21]. It is commonly agreed that the high barn temperature might increase the temperature of both surface and manure and thus lead to increased emissions [10, 21]. Emission increases considerably when the weather is warming up and tending to be drier [17]. Nonetheless, the researchers determined that the direct effects of temperature on ammonia emissions differ significantly. Ammonia emission remains of about two times lower at manure temperature of some 15°C if compared to that at 25°C[22]. Exponential increase of ammonia emission close to three times, as well as increasing the CO_2_ and CH_4_ emission, was observed at the temperature increasing from 5°C to 35°C in the test chambers [23]. Ammonia emission varied from 11 to 88 g per cow per day when air temperature changed from 2.3°C to 22.4°C in naturally ventilated cowshed [24, 25]. Temperature frequently remains the main reason for lower quantities of gas pollutant which are emitted from the open cowsheds than those from the insulated barn. According to Teye [13], a cold barn featured temperature fluctuates from −7°C to +24°C, and ammonia emission ranges from 7 g to 35 g per day per cow. Different scientific studies are carried out generally at different temperatures, temperature ranges, and different chemical composition of manure; thereafter the results are rather different; for example, an increase in temperature of 1°C results in ammonia emission increase of 10% to 39%. Most of the researches are carried out on ammonia by analysing emissions from manure at various temperatures.

In order to find the optimal inner temperature in the cowsheds which ensures that the animals are not harmed and to relate it to the emissions from naturally ventilated open cowsheds, the research focused on (i) gathering the reliable data on ambient temperature and ammonia emission process across different cowsheds and (ii) analysing the effects of the temperature gradient on the ammonia emissions.

## 2. Materials and Methods 

### 2.1. Microclimate Measurements in Cowsheds

Microclimate pilot studies were carried out on the cowsheds of the three types prevailing in Lithuania: semideep insulated in the Training Farm of Aleksandras Stulginskis university (Kaunas distr.), cold in the cooperative Lumpėn*ų* rambynas (Lumpėnai, Šilutė distr.), and box partially thermally insulated (insulated only barn roof) in the company Bernatoniai (Bernatonys, Kaunas distr.). A semideep barn is often a result of the reconstruction of the old small tied barn. Some 140 cows are kept in the semi deep barn, which features its walls being built of concrete blocks and floor (ceilings) insulated with a thick layer of straw. Barn is equipped with the channel ventilation system. The surface of cow places is littered with straw (four rolls of 350 kg of straw litter per day). The mobile manure technology is applied once per month. The box cold barn contains 220 and has uninsulated wall and roof. Its roof is only capped with tin plate, the average wall, and roof heat transmission coefficient which equals 4.5 W (m^2^ K)^−1^. The cows are kept in shallow boxes, floors of which are covered with rubber mats 30 mm in thickness. Walking tracks are covered in concrete, whereas the manure is removed by a scraper transporter. The cowshed is equipped with a nonchannel, ridge-slit ventilation system. The air inflows through wall slots covered with grids and outflow are removed through the ridge holes. Air circulation was controlled by lifting the securer blind and changing width of wall slots. The average wall and roof heat transmission coefficients were 3.3 W (m^2^ K)^−1^ and 0.45 W (m^2^ K)^−1^, respectively, in the partially insulated (insulated roof only) box-type cowshed of 230 boxes. The fresh air inflows occurred through the securer adjustable onwall openings, whereas the contaminated one is removed through a regulated ridge slots. The no-littered technology is applied in the cowshed. The rubber covers are laid in the rest boxes. The manure tracks were covered with grid. 1.2 m deepness manure circulation channels were installed under the cows walking tracks.

The key microclimate variables (temperature and ammonia concentration) were measured in the cowsheds at different seasons of the year. Air temperature was recorded every hour with a computer-controlled temperature and humidity meter-storage device COX TRACER Almemo 2590-9. Preset temperature measuring range recorded was from −30°C to 40°C (±0.3°) with 8 sensors, two outer and six inner at various locations. Ammonia gas concentration in the cowsheds was measured using air sampling system ECOM which facilitated air sampling and transportation to the laboratory, whereas the NH_3_ concentration was measured by the analyser GME700.

### 2.2. Research in Laboratory Experimental Bench

**Figure 1:**
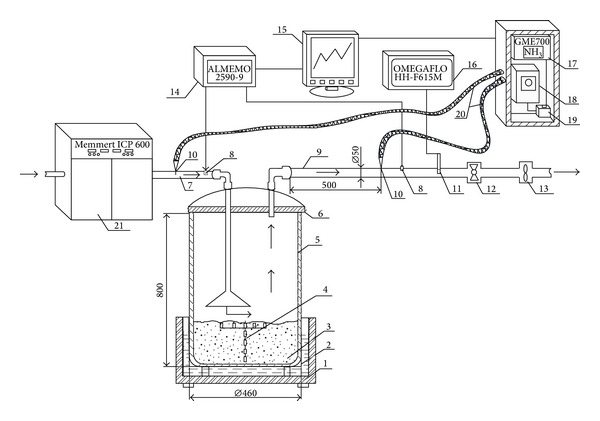
A test bench for modelling the gas emissions from manure: 1: thermostat, 2: water, 3: emission source-manure, 4: thermistor, 5: tight/close manure chamber (100 L capacity), 6: cover, 7: air supply duct, 8: the temperature and humidity sensors, 9: the outflow air duct, 10: air sampling probe, 11: thermoanemometer sensor, 12: valve, 13: a fan with frequency converter, 14: meter-logger ALMEMO 2590-9; 15: PC (program AMR), 16: anemometer OMEGAFLO HH-F615 M, 17: laser gas analyser GME700 18: electrically heated three-channel valves; 19: diaphragm air Pump, 20: heated air hose, 21: climatic camera Memmert.

Having defined the methodological problems of gases analysis in the open naturally ventilated cowsheds, an experimental bench (Figure 1) for modelling of the emission processes from manure under varying ambient temperature was designed and produced. The impact temperature on ammonia emission intensity from manure was determined in the test bench by modelling potential temperature in cowsheds. The fresh barn manure (3) was placed into the chamber (5) in a layer of 0.12 to 0.15 m. The manure chamber was placed on the thermostat (1), which heats the manure from the bottom. Two ducts entered into the chamber: the warmed air from the climatic chamber Memmert ICP 600 (21) through duct (7) was imputed to the manure chamber (5) and was pumped out through the second duct (9) of 50 mm diameter and 1500 mm length. The input duct length of the air sampling probe was 500 mm, that is, 10 times larger than its diameter. Such duct length ensured a laminar air flow.

Air flow velocity in the duct and chamber ventilation intensity was adjusted by using the frequency converter to adjust the fan (13) rotation and thus valve (12) to change the duct diameter area. Air velocity was measured in duct (9) by anemometer OMEGAFLO HH-F615 M and was converted to air flow intensity. The temperature and humidity of the outflow-inflow air of the chamber were measured by the temperature and humidity sensors (8) of the system ALMEMO 2590-9 (14). Gas emission intensity from the manure was measured by applying the mass flow method. The emission intensity (*E*) was calculated into account, the chamber ventilation intensity *G* (m^3^ h^−1^), gas concentration in the outflow (Ce, mg m^−3^), and gas concentration in the inflow air (Co),

(1) $$\begin{array}{c} E=\left(C_{o}-C_{e}\right)G. \end{array}$$

The ammonia concentration was measured by the means of the gas analyzer GME700. Air samples from the ducts (9 and 7) were taken by probe (10) and through heated hose (20) supplied to the gas analyser (17). The air was supplied to the analyser continuously by pump (19) with a capacity of 6 l min^−1^. To prevent the condensation of the air, it was warmed to 150°C in intestine (20) and electrically heated valve (18). The ammonia gas analyser GME700 determined the gas concentration in the air by the laser spectroscopy. Ammonia concentration was measured continuously and recorded every 1 min

The temperatures of manure and its surface were measured by employing thermocouples (4) with a wire diameter of 0.1 mm. Three thermocouples were laid on the manure surface and sensors of the other remaining thermocouples were arranged in vertical straight line at various manure depths. Temperature measurements were recorded by ALMEMO 2590-9 with a microprocessor for data processing and logger system. The sensors were connected to device ALMEMO featuring 9 inputs (ZA9020-FS) suitable for copper-constantan thermocouple. The fresh cattle manure of the varying moisture was assessed. The mixed and homogenised manure from the barn was taken in 20 litre buckets, which were placed for 24 hours in climate chambers with different temperatures: 4.2°C, 13.1°C, 24.5°C, and 35.6°C. Then manure has been spilled from the bucket in 0.12 m thickness layer in the bench chamber and stirred and the ammonia emission intensity was measured. During testing, temperature of supplied 3.26 ± 0.21 m^3^ h^−1^ air flow to the manure chamber was 18.6 ± 0.4°C, while manure from the bottom has not been heated. These emission tests lasted 70 min; 2 tests were performed with manure of different content of dry matter (DM): 11.84 ± 0.12%, and 8.62 ± 0.09%.

### 2.3. NH_3_ Emission of Heated Manure

In order to determine the detailed influence of temperature on ammonia emissions from the manure (12.45 ± 0.11% DM), the manure chamber (5) was heated (45°C) from the bottom by the thermostat (1) generating temperature gradient in the vertical layers of the manure. The chamber temperature was constant at 20.6 ± 0.3°C. To determine temperature effect on ammonia emissions, the inflow air was heated by 6-7°C periodically every 20 hours. The test lasted 120 hours.

### 2.4. Statistical Analysis

The confidence intervals of the estimates were obtained by employing one-way analysis of variance (by *ANOVA*). The least significant differences between treatment means were determined using Fisher's least significant differences. Standard error (SE) has been calculated at a level of statistical significance *P* < 0.05.

## 3. Results and Discussion 

### 3.1. Temperature Variation in Different Cowsheds

**Figure 2:**
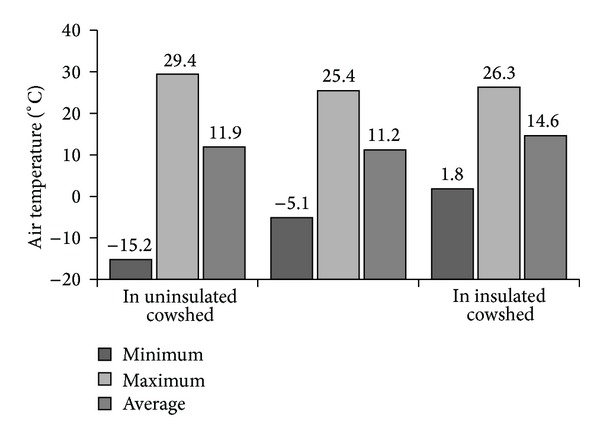
Inner temperature in different cowsheds in Lithuania (*P* < 0.05).

In order to meet the cowsheds development trends, the research was performed in the most common cowshed type in Lithuania. Microclimate tests were performed during various seasons in three cowshed types: semideep, cold box, and box partially thermally insulated (insulated roof only). Animal keeping technology, the design of the construction, types and quantities of accumulated manure, and other environment factors varied across the cowsheds. Thereafter, they were specific with different inner temperature, one of the main features determining their functionality, affecting microclimate and gas emission from manure in cold cowsheds. During microclimate trials, the outer temperature ranged in between −21.5°C and 32.0°C (Figure 2). Nonetheless, the inner temperature differed considerably: fell to −15.2°C and only 1.8°C in cold and semideep cowsheds, respectively. The highest temperature of 29.4°C was observed in the cold box cowshed indeed. Throughout the year, semideep barn remained the warmest. Specifically, average annual temperature of 14.6°C was observed there, whereas slightly lower mean temperatures of 11.9°C and 11.2°C were observed in cold and partially insulated boxes, respectively.

These data were applied to estimate the regression equations relating air temperature in different cowsheds to the outer temperature (Table 1). Correlation coefficient (*r* = 0.9) shows a strong positive correlation between outer and inner temperatures of different cowsheds.

Different ambient temperature resulted in a varying ammonia concentration across various cowsheds during different seasons. When temperatures fall below 0°C, ammonia was not found in cold box cowshed. It was determined that the warming weather and rising inner temperature increased ammonia concentration in barn. According to Zhang et al. [20], the warm and moist conditions not only promoted the spread of disease but also facilitated favourable conditions for ammonification bacteria activity and, thus, for ammonia emissions from manure. When temperature rose above 20°C in the barn, ammonia concentration increased to 8.5–9.0 ppm and from 13.5 to 14.8 ppm in semi deep barn due to lack of barn ventilation.

### 3.2. Modelling of Ammonia Emission under Changing Temperature in the Laboratory Test Bench

**Figure 3:**
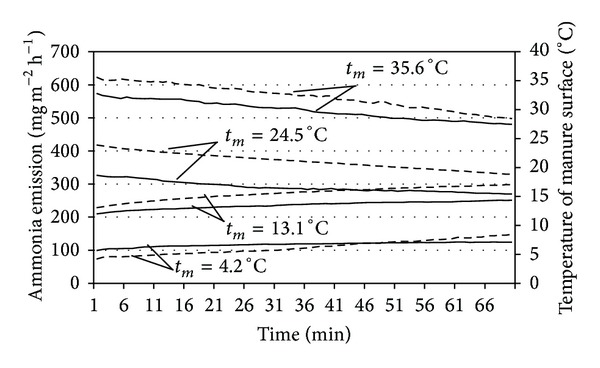
Ammonia emission (solid line) intensity from cattle manure at changing surface temperature (dashed line), *t*_*m*_: manure inner temperature.

As fluctuating temperature transformed air circulation, the determination of the effect of barn temperature on ammonia emission was rather cumbersome. For that reason, the impact of the temperature on ammonia emissions from manure was assessed in the laboratory test bench (Figure 1). The potential impact of air and manure surface temperature as well as temperature gradient of manure layers on ammonia emission intensity was determined by modelling temperature in cowsheds during different seasons. Dry matter contents in fresh manure of 11.84 ± 0.12% and 8.62 ± 0.09% were recorded of the first and second test, respectively. It was found out that manure temperature affected ammonia emission (Figure 3). The manure surface temperatures and ammonia emission intensity is changing towards trend. Emissions were recorded under the two temperature conditions: (1) a higher-temperature inflow which warmed the manure thus increasing ammonia emission and (2) a lower-temperature inflow temperature which was lower if compared to the temperature of manure. Ammonia emission significantly increased at the higher temperatures of layer the manure. NH_3_ emissions of 323 ± 2.8 mg m^−2^ h^−1^ and 565 ± 5.9 mg m^−2^ h^−1^ were recorded at 24.5 ± 0.39°C and 35.6°C manure temperatures, respectively.

Ammonia emission increased almost sixfold, while manure temperature increased from 4.2°C up to 35.6°C; that is, the temperature increase of 1°C increased the ammonia emission by 14.7 mg m^−2^ h^−1^ on average. In addition, this is equivalent to 74.09 g m^−2^ per indoor period (210 day) or some 259 g per cow space.

When inflow temperature was lower than that of manure, the manure was being cooled, temperature falling of its surface, leading to reduction in ammonia emissions. NH_3_ emission (103 ± 2.4 mg m^−2^ h^−1^ and 214 ± 3.2 mg m^−2^ h^−1^ resp.) was observed only at manure temperatures of 4.2 ± 0.12°C and 13.1 ± 0.29°C which were lower than that of inflow air. The impact of temperature on the emission of ammonia at temperatures of 4°C to 36°C is considerably different; therefore it is appropriate to analyse these processes separately.

**Figure 4:**
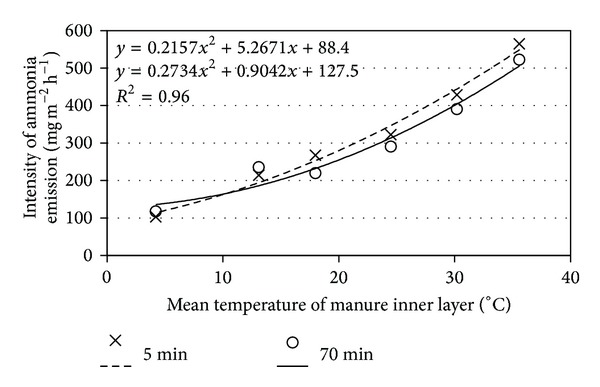
Mean intensity of ammonia emission from fresh cattle manure during 5 and 70 min in against temperature of the inner layer.

Ammonia emission increased exponentially with the increasing manure temperature (Figure 4). This dependence remained similar within both short (5 min) and longer (70 min) periods. The stronger impact of manure temperature on ammonia emission intensity was observed in a short period as well as at higher temperatures. Indeed, the increase in manure temperature of 1°C causes the ammonia emission to increase by 7.3 mg (m^2^ h)^−1^, 10.5 mg (m^2^ h)^−1^, and 17.4 mg (m^2^ h)^−1^ at 10–20°C, 20–30°C, and above 20°C temperatures, respectively. Subsequently, the temperature rise above 20°C must be avoided.

**Figure 5:**
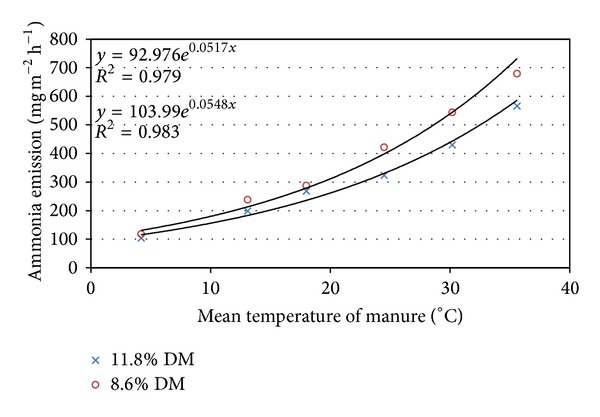
Intensity of ammonia emission from cattle manure of different consistency (DM) in dependence on its inner temperature.

Analysis of smoother manure of less dry matter content (8.62% ± 0.09%) revealed similar correlations with temperature (*r* = 0.9; Figure 5). Ammonia was dispensed more intensively from smoother manure, with increasing emission gains at the higher temperatures.

**Figure 6:**
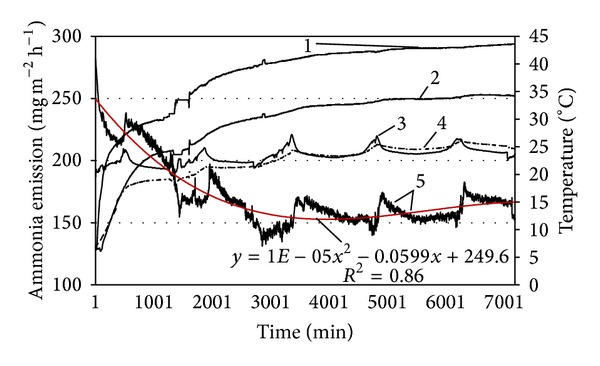
Impact of air and manure temperature on the intensity of ammonia emission from cattle manure. 1: manure temperature of the bottom layer; 2: manure temperature in the middle layer; 3: manure surface temperature; 4: air temperature; 5: ammonia emission.

In order to analyse manure temperature's impact on ammonia emission in a more detailed way, the manure was heated from the bottom up to 45°C. The manure temperature rose from 6.1°C to 43.6°C in the bottom layer, while only to 24.6°C in manure surface during the testing period (Figure 6). Intensity of ammonia emission was rather volatile one: it has decreased from 285 mg m^−2^ h^−1^ down to 130 mg m^−2^ h^−1^ during the 50 hours of research and then started to increase as a result of the rising temperature of the manure surface. What the curves of ammonia emission intensity and manure temperature in various layers do indicates that relation between ammonia emission and manure temperature remained rather arbitrary.

**Figure 7:**
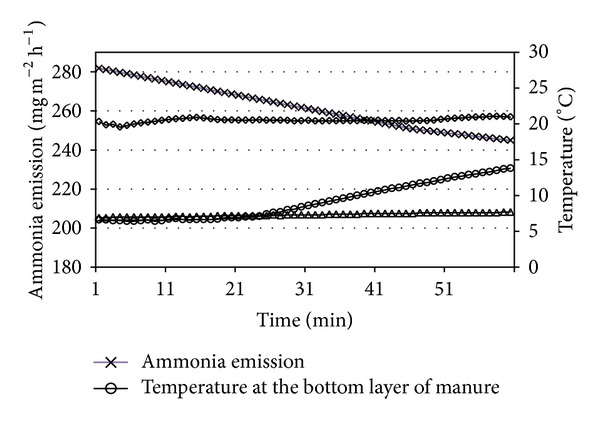
Ammonia emission intensity in relation with temperature variation of air and manure.

Ammonia emission is mainly determined by variations in the temperatures of air and manure surface. Increases in the temperature of the airflow by several degrees centigraded render increase in the temperature of the manure surface and, subsequently, increase in ammonia emission. The analysis of ammonia emission from manure can be analyzed in terms of the short and long periods. Analysis of the processes which occurred during the first 60 min (Figure 7) indicated that the heat source at the bottom of manure layer did not affect the manure surface temperature, as the temperature did not increase in the inner layer of manure. Indeed, the ammonia emission decreased by some 13%, though temperature rose by only 1.1°C in manure surface during the first 60 min.

Nonetheless, the observed values of the ammonia emission were different from those reported in other researches [10, 21, 22]. These differences might have been caused by divergent tests conditions and chemical composition of manure. Anyway, the determined temperature impact on ammonia emissions from manure is adequate to the results obtained in many researches [13, 17, 20, 23, 25]. Therefore the obtained relationships provided possibilities to analyse and evaluate the processes of ammonia emissions from manure amidst the varying ambient temperature: inner and surface manure temperatures as well as air temperature. Hence, temperature inside the manure remained an important factor as long as it affected both the surface temperature of manure and underlying processes of the manure surface, for example, drying and crust formation. It was found out that the intensity of these processes generally depends on the difference between the air and manure temperatures. Ammonia emissions from manure can be considerably reduced by controlling the temperature of the air and manure surface. This is explained by the fact that the manure surface's temperature has a significant effect on activity of aerobic ammonification bacteria [26].

## 4. Conclusion 

Air temperature in cowsheds varied within a wide range of 15°C to 30°C during different seasons of the year. Therefore, volatile conditions for ammonification bacteria activity prevailed and thus affected ammonia emissions from manure. The following two issues remain essential in terms of reduction of ammonia emissions from the livestock buildings: First, the optimal regulation of the air temperature in cowshed, especially at high temperatures. Second, the management of the airflow preventing the warm ambient air from blowing directly into the manure. Reduction of the average annual air temperature by 1°C in cowshed declined the annual ammonia emissions by some 5.0%.

The trials carried out in the laboratory test bench revealed that the key factor inducing ammonia emissions is temperature of the manure surface and the ambient temperature, which, indeed, directly affected the manure surface's temperature. With increasing manure temperature, ammonia emission increased in exponential way. Thus, the latter's effect was greater at higher temperatures. Specifically, increase in the manure temperature from 4°C to 30°C caused growth in ammonia emission from 102 mg m^−2^ h^−1^ to 430 mg m^−2^ h^−1^, namely, about 4 times. All in all, the temperatures above 20°C should be avoided, for temperature rise by 1°C in this region caused increase of ammonia emission of more than 17 mg m^−2^ h^−1^. The analysis of emission processes from the manure has to involve assessment of air and manure surface's temperatures, as well as the temperature's gradient. The inner temperature of the manure layer remains important as long as it affects both its surface temperature and the underlying processes in manure surface, for example, drying and crust formation.

## Figures and Tables

**Table 1:** Correlation between inner, *t*_*c*_, and outer, *t*_*o*_, temperatures in different cowsheds.

Cowshed type	Regression
Cold box	*t*_*c*_ = 0.865*t*_*o*_ + 5.17; *R* ^2^ = 0,980 (*P* < 0.05)
Box partially thermally insulated	*t*_*c*_ = 0.5039*t*_*o*_ + 7.03; *R* ^2^ = 0.934
Semi deep insulated	*t*_*c*_ = 0.4462*t*_*o*_ + 11.78; *R* ^2^ = 0.822
